# Lack of evidence for xenotropic murine leukemia virus-related virus(XMRV) in German prostate cancer patients

**DOI:** 10.1186/1742-4690-6-92

**Published:** 2009-10-16

**Authors:** Oliver Hohn, Hans Krause, Pia Barbarotto, Lars Niederstadt, Nadine Beimforde, Joachim Denner, Kurt Miller, Reinhard Kurth, Norbert Bannert

**Affiliations:** 1 Robert Koch-Institute, Centre for Biological Safety 4, Nordufer 20, 13353 Berlin, Germany; 2 Charité - Universitätsmedizin Berlin, Urologische Klinik, Schumannstraße 20/21, 10117 Berlin, Germany; 3 Charité - Universitätsmedizin Berlin, Hindenburgdamm 30, 12203 Berlin; 4 Robert Koch-Institute, Retrovirus induced immunosuppression (P13), Nordufer 20, 13353 Berlin, Germany

## Abstract

**Background:**

A novel gammaretrovirus named *xenotropic murine leukemia virus-related virus *(XMRV) has been recently identified and found to have a prevalence of 40% in prostate tumor samples from American patients carrying a homozygous R462Q mutation in the RNaseL gene. This mutation impairs the function of the innate antiviral type I interferon pathway and is a known susceptibility factor for prostate cancer. Here, we attempt to measure the prevalence of XMRV in prostate cancer cases in Germany and determine whether an analogous association with the R462Q polymorphism exists.

**Results:**

589 prostate tumor samples were genotyped by real-time PCR with regard to the RNaseL mutation. DNA and RNA samples from these patients were screened for the presence of XMRV-specific *gag *sequences using a highly sensitive nested PCR and RT-PCR approach. Furthermore, 146 sera samples from prostate tumor patients were tested for XMRV Gag and Env antibodies using a newly developed ELISA assay. In agreement with earlier data, 12.9% (76 samples) were shown to be of the QQ genotype. However, XMRV specific sequences were detected at neither the DNA nor the RNA level. Consistent with this result, none of the sera analyzed from prostate cancer patients contained XMRV-specific antibodies.

**Conclusion:**

Our results indicate a much lower prevalence (or even complete absence) of XMRV in prostate tumor patients in Germany. One possible reason for this could be a geographically restricted incidence of XMRV infections.

## Background

Prostate cancer (PCa) is currently the most commonly diagnosed cancer in European males and causes approximately 80,000 deaths per year [1]. Modern methods of diagnosis and extensive programs for early detection have increased the chances for successful treatment in recent years, but there is still only limited knowledge concerning susceptibility and putative risk factors for PCa. In addition to age, the risk factors for developing PCa are thought to be diet, alcohol consumption, exposure to ultraviolet radiation [2], and genetic factors [3]. One of the first studies to investigate the hereditary factors associated with a predisposition for developing prostate cancer identified the HPC1 locus (hereditary prostate cancer locus-1) [4], which is now known to harbor the RNaseL gene. RNaseL codes for a endoribonuclease involved in the IFN-regulated antiviral defense pathway (reviewed by [5]). The significance of RNaseL gene polymorphisms for the development of prostate cancer is still under scrutiny. The R462Q (rs486907) polymorphism for example is implicated in up to 13% of all US prostate cancer cases [6] and three other variants contribute to familial prostate cancer risk in the Japanese population [7], whereas no significant association with disease risk could be found in the German population [8].

Recently, an analysis for viral sequences in prostate cancer stroma tissues using custom-made microarrays resulted in the discovery of a new gammaretrovirus named *xenotropic murine leukemia virus-related virus *(XMRV), [9,10]. XMRV was present in eight of twenty (40%) cases in patients with familial prostate cancer that were homozygous at the R462Q locus for the QQ allel. On the other hand, the virus could be detected in only 1.5% of carriers of the RQ or RR allels. In subsequent studies involving smaller cohorts of European prostate cancer patients, the prevalence and correlation of the QQ-phenotype with the presence of XMRV were either far less significant [11] or the virus could not be detected at all [12]. Very recently XMRV was recognized by immunohistochemistry in 23% of prostate cancers from US American donors, independent of the R462Q polymorphism [13].

This present study describes the development and use of sensitive PCR and RT-PCR assays to test DNA and RNA from 589 PCa tumor samples obtained from the Charité hospital in Berlin (Germany) for the presence of proviral XMRV DNA and corresponding viral transcripts. In addition, we used an ELISA based on recombinant XMRV proteins to screen 146 PCa patient sera for viral Env- and Gag-specific antibodies. Neither in the 76 specimens homozygous for the QQ allele, nor in any of the other samples could XMRV or a related gammaretrovirus be detected. Furthermore, none of the sera contained antibodies specific for the XMRV Env or Gag proteins.

## Methods

### Patients

Tissue samples were collected from 589 patients undergoing radical prostatectomy for histologically proven primary prostate cancer at the Department of Urology, Charité - Universitätsmedizin Berlin, between 2000 and 2006. Institutional review board approval for this study was obtained and all patients gave their informed consent prior to surgery. Tissue samples were obtained immediately after surgery, snap-frozen in liquid nitrogen and stored at -80°C. Histopathologic classification of the samples was based on the World Health Organization and 1997 TNM classification guidelines (International Union Against Cancer, 1997). The patient's median age was 63 years (range 43 - 80). The serum PSA levels were measured prior to surgery and ranged from 0.1 to 100 ng/ml (median 7.5 ng/ml). 405 of 589 patients (69%) had organ-confined disease (pT2) while the remaining 31% had non organ-confined disease (pT3 and pT4). Using the Gleason-score (GS) system, the sample population was divided into low-grade tumors (GS 2-6, n = 282), intermediate cases (GS 7, n = 175), and high-grade prostate carcinomas (GS 8-10, n = 68).

### Nucleic acid isolation

Frozen tissues were mechanically sliced and immediately lysed in DNA- or RNA-lysis buffer, column-purified, and eluted (50-200 μl) according to the manufacturers instructions (QIAamp DNA Mini Kit, RNeasy Mini Kit, QIAGEN GmbH, Hilden, Germany). The OD_260/280 _ratio and nucleic acid concentrations were determined using the Nanodrop-1000 instrument (PeqLab Biotechnologie GmbH, Erlangen, Germany). In addition, RNA samples were checked for integrity using a Bioanalzyer-2100 (Agilent Technologies, Inc., Santa Clara CA, United States). No additional macro-/micro-dissections were performed on the prostate tissues because viral nucleic acids were expected to be present preferentially in the stromal compartments.

### Diagnostic PCR

A nested PCR was developed for the detection of XMRV sequences that amplifies regions upstream of the *gag *start codon, harboring the unique 24 nt deletion [10]. First, we constructed by fusion-PCR a synthetic gene representing the region from nucleotide 1 to 800 of the MLV DG-75 (Genbank acc. number AF221065). This fragment was cloned into the pCR4-TOPO vector (Invitrogen, Karlsruhe, Germany), and the identity of the fragment was confirmed by sequencing. The same procedure was used to clone a corresponding 800 nt fragment for use as a positive control of the XMRV genome (Genbank acc. num. EF185282). Conditions of first round PCR for the detection of proviral sequences were: 100 ng patient DNA, primer Out-For 5'-CCGTGTTCCCAATAAAGCCT-3', Out-Rev 5'-TGACATCCACAGACTGGTTG-3', (30 sec @ 94°C, 30 sec @ 60°C, 30 sec @ 72°C) × 20 cycles. Using 1/10^th ^of the first reaction and primer In-For 5'-GCAGCCCTGGGAGACGTC-3' and In-Rev 5'-CGGCGCGGTTTCGGCG-3' the second round PCR is able to detect any XMRV-like sequences, e.g. MLV DG-75. In addition, using a primer spanning the XMRV-specific deletion 5'-CCCCAACAAAGCCACTCCAAAA-3' we were able to distinguish between XMRV and DG75 sequences. Second round PCR reaction was performed at an elevated annealing temperature of 64°C for 35 cycles.

A nested-PCR strategy was used to detect XMRV-specific viral RNAs in the total RNA of prostate tissue samples in which the first round RT-PCR was performed as described above using *In-For *and *In-Rev *followed by a quantitative real-time PCR published by Dong *et al*., 2007 [9,14]. As an internal control, a human GAPDH specific primer and probe set were included in which the primers for the outer RT-PCR were the same as for the inner PCR: forward 5'-GGCGATGCTGGCGCTGAGTAC-3' reverse 5'-TGGTCCACACCCATGACGA-3' and the probe 5'-YAK-TTCACCACCATGGAGAAGGCTGGG-Eclipse Dark quencher-3' [15].

### RNaseL genotyping

A real-time PCR setup designed by Olfert Landt/TIB MOLBIOL, Berlin was used for RNaseL genotyping of tumor samples which detects the single nucleotide polymorphism G1385A (rs486907) responsible for the R462Q mutation. PCR was carried out with R462Q_F CCTATTAAGATGTTTTGTGGTTGCAG, R462Q_A GGAAGATGTGGAAAATGAGGAAG and the probes R462Q_(A) YAK-ATTTGCCCAAAATGTCCTGTCATC-BBQ and R462Q_(G) FAM-ATTTGCCCGAAATGTCCTGTCATC-BBQ following a two-step protocol with 95°C for 20 sec and 60°C for 1 min. Positive controls were constructed by fusion PCR, starting with 40 mer oligonucleotides, of the two 297 bp fragments corresponding to the "R"- and "Q" versions of the RNAseL genomic region and cloning these into the pCR4-TOPO vector (Invitrogen). For each PCR, positive control plasmids containing the R- or Q-sequence were included.

### Recombinant Proteins, Immunization

Recombinant proteins of XMRV pr65 (Gag) and gp70 (Env/SU) were generated for immunization and for the ELISA assays. For XMRV Env/SU, a fragment containing the amino acids 1-245 of the surface unit (gp70) was amplified, cloned in pET16b vector (Novagen, Gibbstown, USA) and expressed in BL21 *E. coli*. For XMRV Gag (pr65), two fragments (amino acids 1-272 and 259-535) that overlap by 14 amino acids were constructed. The expressed proteins were affinity purified using a Ni-NTA column and eluted in 8 M urea, and proteins for immunization were subsequently dialyzed against phosphate buffered saline. BALB/c mice were immunized with the recombinant fragments of the Envelope or Gag proteins, and sera were collected throughout the period of four immunizations. All animal experiments were performed in accordance with institutional and state guidelines.

### ELISA

Two weeks after the last immunization the mice were bled, and serum antibodies were measured by solid phase enzyme-linked immunosorbent assay (ELISA). Briefly, bacterially expressed and purified (via His-tag) protein fragments were coated overnight on Probind-96-well plates (Becton Dickinson Labware Europe, Le Pont de Claix, France) at room temperature in equimolar amounts. The plates were blocked with 2% Marvel milk powder in phosphate buffered saline (PBS) for 2 h at 37°C, washed three times with PBS, 0.05% Tween 20 and serial diluted mouse sera or patient sera at a 1:200 dilution in PBS with 2% milk powder and 0.05% Tween20 added into each well. After incubation for 1 hour at 37°C, each well was again washed three times and a 1:1000 dilution of a goat anti-mouse IgG-HRP conjugate (Sigma Aldrich, Munich, Germany) in PBS, 2% milk powder, 0.05% Tween 20 (Serva, Heidelberg, Germany) was added. After further incubation for 1 hour at 37°C, each well was again washed three times. The chromogen ortho-phenylendiamin (OPD) in 0.05 M phosphate-citrate buffer, pH 5.0 containing 4 μl of a 30% solution of the hydrogen peroxide substrate per 10 ml was then added. After 5-10 minutes the color development was stopped by addition of sulphuric acid and the absorbance at 492 nm/620 nm was measured in a microplate reader.

Patient sera were tested for XMRV-Gag or -Env binding antibodies in the same way, using a goat anti-human IgG-HRP conjugate as secondary antibody (Sigma Aldrich, Munich, Germany). Out of the 146 sera samples only from 30 patients the corresponding nucleic acids were included in the 589 DNA/RNA samples.

### Immunofluorescence microscopy

Cells were grown on gelatine (0.3% coldwater fish gelatine in distilled water) coated glass slides in 12-well plates and 24 h after seeding were transfected using Polyfect Reagent (Qiagen) with the full length molecular clone pCDNA3.1-VP62 or with the pTH-XMRV-coEnv or pTH-XMRV-coGAG plasmids containing codon optimized synthetic full-length genes of the XMRV *env *or *gag *under control of the CMV promoter. 48 h after transfection the cells were fixed with 2% formaldehyde (Sigma) in PBS. Cells were rinsed briefly in PBS, permeabilized with 0.5% Triton X-100 in PBS for 15 min and washed 3 times with PBS. After 30 min incubation with blocking buffer (2% Marvel milk powder in PBS) cells were incubated for 60 min at 37°C with the mouse or human antisera diluted 1:200 in blocking buffer. The slides were washed extensively with PBS. The secondary antibodies conjugated to fluorophores were added for 30 min. After thorough washing steps with PBS, the cells were mounted in Mowiol and the glass slides were placed upside-down on microscopy slides. Images were obtained on a Zeiss (LSM510) confocal laser-scanning microscope.

### Electron Microscopy

Transfected cells were fixed with 2.5 % glutaraldehyde in 0.05 M Hepes (pH 7.2) for 1 h at room temperature. Fixatives were prepared immediately before use. The samples were embedded in epoxy resin (Epon) after dehydration in a series of ethanol solutions (30%, 50%, 70%, 95%, and 100%) and infiltrated with the resin using mixtures of propylene oxide and resin followed by pure resin. Polymerization was carried out at 60°C for 48 h. Ultrathin sections (60-80 nm) were cut with an ultramicrotome (Ultracut S or UCT; Leica, Germany) and picked up on slot grids covered with a pioloform supporting film. To add contrast, sections were stained with uranyl acetate (2% in distilled water) and lead citrate (0.1% in distilled water). Sections were examined with a FEI Tecnai G2 transmission electron microscope.

## Results

### Determination of the RNaseL genotype of prostate cancer samples

**Figure 1:**
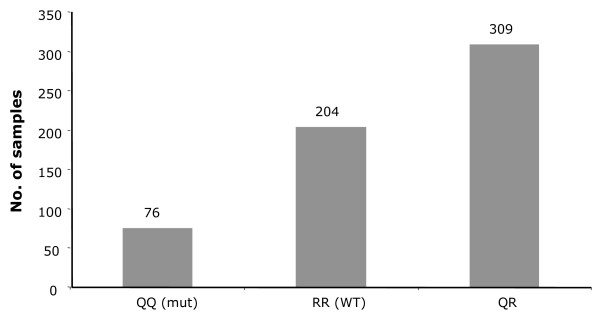
**Analysis of sample DNA with allele specific real time PCR for the R462Q genotype**. 76 of 589 samples (12.9%) are homozygous for the QQ allele, 204 samples (34.6%) are homozygous for the RR allele and 309 (52.5%) are heterozygous.

The highly significant correlation between XMRV-positive prostate cancers and homozygosity for the QQ allel of the RNaseL SNP R462Q previously published [9,10] prompted us to analyze the genotypes of all 589 PCa samples included in our study. The DNA was extracted from prostate biopsies consisting of tumour cells and surrounding stromal tissue. Using a real-time PCR method that allows the underlying G1385A mutation at the DNA level to be detected, 76 specimens (12.9 %) were found to be homozygous for the QQ genotype. The RQ and RR genotypes were present at frequencies of 52.5% and 34.6% respectively (Fig. 1). All samples were screened in duplicate and gave consistent results.

### Screening for proviral XMRV sequences by nested PCRs

**Figure 2:**
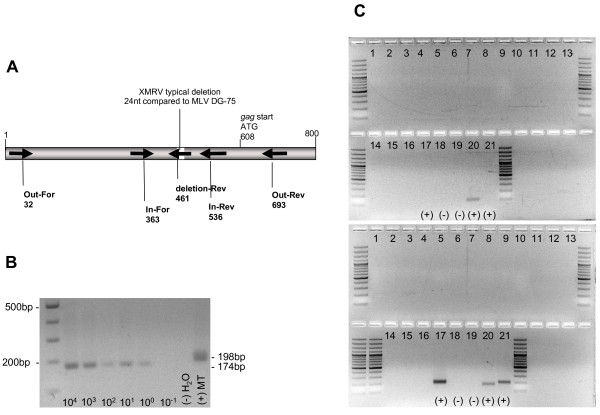
**Nested PCR for sensitive screening of patient tumor tissue DNA**. **(A) **A nested PCR primer setup was used as indicated for the screening of 589 PCa patient DNA isolated from prostate tumor and stroma tissue. Primer sites are numbered according to the XMRV VP62 sequence (Genbank EF185282). **(B) **The reproducible detection limit was 10 copies of plasmid DNA in human genomic DNA resulting in a 174 bp PCR product for XMRV. In the experiment shown even 1 copy could be amplified. Mouse tail DNA (MT) was used as positive control yielding a 198 bp product amplified from endogenous genomic MLV sequences. **(C) **Nested-PCR screen of the first 16 QQ patients (lane 1-16) with the In-For and Deletion-Rev primer pair (upper panel) and In-For and In-Rev primer setup (lower panel); lane 17 = mouse tail DNA, lane 18 = water control outer PCR mix, lane 19 = water control inner PCR mix, lane 20 = pXMRV, lane 21 = pDG75, marker = 100 bp marker.

We developed a nested PCR able to detect and discriminate between XMRV and proviral sequences closely related to the endogenous murine gammaretrovirus DG-75 [16]. This discrimination is based on the XMRV-specific 24 nt deletion within a conserved retroviral region (Fig. 2A). To facilitate the development of the nested PCR and to evaluate its sensitivity, we constructed *de novo *the corresponding XMRV genomic region (nt 1-800 of the XMRV VP62 sequence) via fusion-PCR of oligonucleotides and cloned this fragment into the pCR4-TOPO vector to generate the pXMRV plasmid. In addition, the corresponding sequence of the DG-75 provirus was assembled and cloned in the same way to yield the pDG-75 vector.

Chromosomal DNA from a healthy human was spiked with serial 1:10 dilutions of pXMRV and used to assess the sensitivity of the nested PCRs. Following the first round that used the outer primers, two parallel second rounds with the primer pair In-For/In-Rev and In-For/Deletion-Rev were performed.

Both primer pairs allowed the specific detection of 10 or more copies of their targets. Use of the primers In-For/In-Rev with the pXMRV template resulted in a 174 nt PCR product, and a 198 nt product was produced with pDG-75 as template (Fig. 2B). Mouse tail DNA was also included as a positive control to amplify a 198 nt sequence from murine endogenous DG-75-like proviruses. As expected, no PCR signal was generated if the In-For/Deletion-Rev primer pair was used with pDG-75 or mouse tail DNA as template (Fig. 2C, lower panel, lane 17 and lane 21).

All 589 DNAs isolated from prostate biopsies were screened using the nested PCR setup and primer combinations described. The successful RNAseL genotyping of all 589 samples confirmed DNA integrity and the absence of PCR inhibitors in the samples. Specific fragments indicating the presence of XMRV (Fig. 2C upper panel) or a DG-75 related gammaretrovirus were obtained from none of the samples (Fig. 2C lower panel).

### Examination of total RNA for the presence of XMRV transcripts

**Figure 3:**
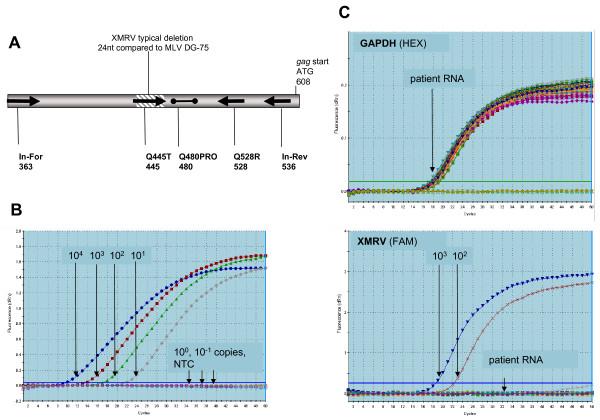
**Nested RT-PCR for sensitive and specific screening of patient tumor tissue RNA**. **(A) **RT-PCR for all 589 RNA samples was carried out with In-For and In-Rev primers, followed by a quantitative real-time PCR using primers and probe as indicated. Using the Q445T forward primer spanning the XMRV typical deletion ensured specific detection of XMRV sequences. Primer sites are numbered according to the XMRV VP62 sequence. **(B) **Real-time PCR curves showing the mean of triplicates. The sensitivity shown in this example was 10 copies. **(C) **Example of the first 16 QQ patients RNA screen including GAPDH control reactions as the mean value of duplicates.

To assess corresponding RNA samples, a comparable approach was used in which a first round RT-PCR for cDNA synthesis with primers amplifying XMRV and DG-75-like sequences was followed by quantitative real-time PCR for the specific detection of XMRV (Fig. 3A). Preliminary experiments performed with XMRV RNA (kindly provided by R. Silverman) indicated the ability to detect as few as 10 transcripts (e.g. Fig. 3B), and the reproducible sensitivity to detect 100 transcripts. A human GAPDH primer and probe set was used in each sample as an internal control for the integrity of the RNA. Whereas all 589 samples generated a positive GAPDH signal with Ct-values between 16 and 20, no signals with the XMRV specific probe were obtained (Fig. 3C).

### XMRV antibody detection

Productive infection of humans by a murine gammaretrovirus related virus should induce an antibody response. Fragments of the cloned XMRV VP62 envelope (gp70) and the gag (pr65) protein were expressed in *E. coli *to provide a basis for an ELISA to detect XMRV-specific antibodies in the sera of prostate cancer patients. One fragment spanning the region from amino acids 1 to 245 of Env and two overlapping fragments spanning Gag were expressed and purified via an N-terminal His-tag.

**Figure 4:**
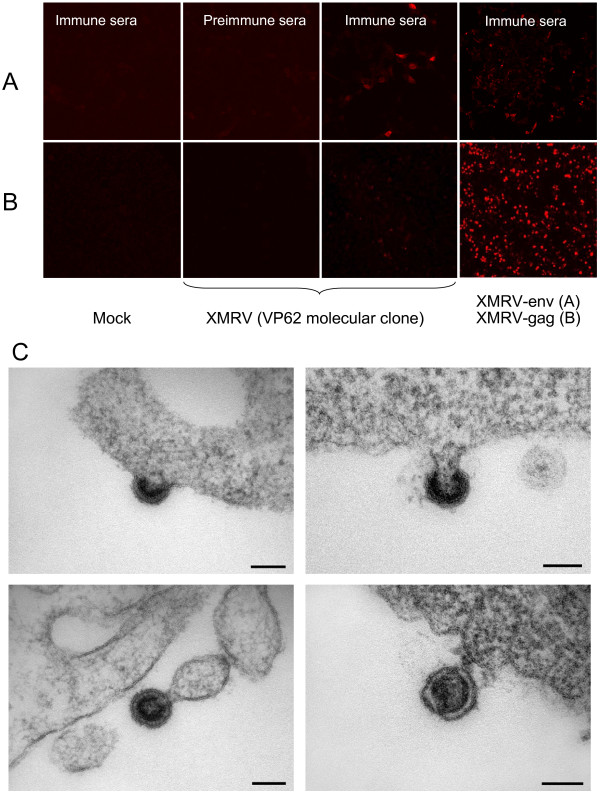
**Immunoflourescence microscopy and electron microscopy of transfected 293T cells**. Mice were immunized with recombinant gp70 or pr65 protein fragments, and sera were used for immunoflourescence microsocopy of 293T cells transfected two days earlier with the molecular XMRV clone VP62 or with gp70 and pr65 expression constructs. **(A) **A pool of sera from gp70 immunized mice showed reactivity against whole XMRV or XMRV envelope protein expressing cells. Preimmune sera showed no binding, and immune sera did not react with naive 293T cells. **(B) **A pool of immune sera from pr65 (Gag) immunized mice showed similar reactivity to whole virus or XMRV Gag expressing cells. Gag protein was expressed at higher levels in cells transfected with the CMV-driven codon-optimized *gag *construct than in those transfected with the VP62 molecular clone of XMRV. **(C) **Thin section of 293T cells 2 days after transfection with the VP62 molecular clone of XMRV. Particles budding at the cell membrane and a mature XMRV virion are shown. Scale bar = 100 nm.

Sera from immunized Balb/c mice (but not pre-immune sera) were reactive in ELISA against the recombinant proteins (data not shown). In addition, the specificity of the antibodies was confirmed by immunofluorescence microscopy using HEK 293T cells transfected with the expression plasmid pcDNA3.1-VP62 (kindly provided by R. Silverman) that carries the sequence of the replication active XMRV molecular clone (Fig. 4A and 4B). After transfection, these cells produce gammaretroviral particles visible by electron microscopy of ultrathin sections (Fig. 4C). This is, to our knowledge, the first visualisation of XMRV particles using thin section electron microscopy of transfected cells. The particles show the typical C-type budding structures and a classical morphology of MLV.

**Figure 5:**
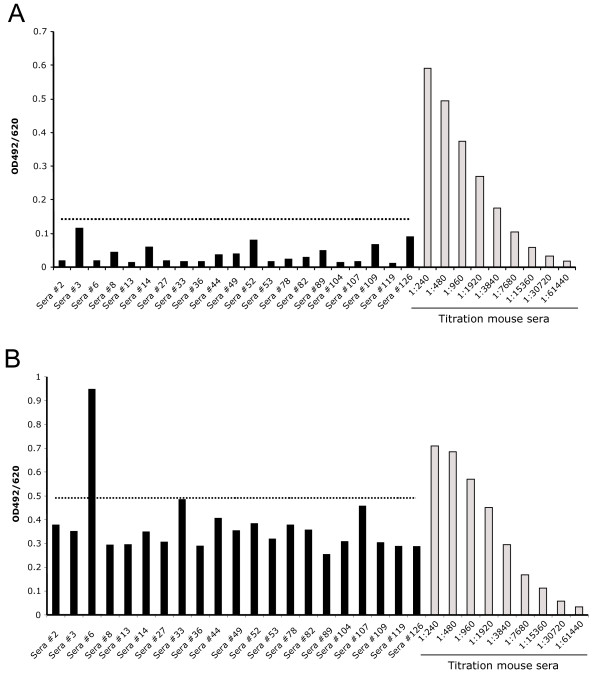
**ELISA of PCa patient's sera using recombinant XMRV proteins**. Mean ODs with two replicates of each patient sera diluted 1:200 (dark bars) and of serially diluted sera from immunized mice (light bars). Cut-off was calculated as the mean of four (gp70) and five (pr65) sera from healthy controls plus two times standard deviation. **(A) **ELISA of randomly chosen PCa patient sera using the gp70 (Env) fragment (aa 1-245). **(B) **ELISA using a mixture of both pr65 (Gag) fragments. In general there was a higher background against the pr65 proteins, seen also with the sera of healthy humans and the preimmune mouse sera.

Of the 146 sera samples tested, the corresponding nucleic acids were available in 30 cases and were included in the amplification reactions as a subset of the 589 DNA/RNA samples. Of these 30 patients 2 were of the QQ genotype, 20 of QR and 8 were RR homozygous. In total, 146 sera from prostate cancer patients and 5 healthy control individuals were tested negative for antibodies binding recombinant XMRV gp70 and Gag proteins in ELISA, although postive control immunized mouse sera reacted strongly (Fig. 5A and 5B). One patient serum that reacted strongly in ELISA against the recombinant pr65 protein was subsequently tested by immunofluorescence assay using HEK 293T cells expressing XMRV and cells expressing the gp70- or pr65 proteins alone. No XMRV specific binding was seen, indicating a non-specific ELISA reaction.

## Discussion

XMRV is a recently discovered gammaretrovirus, found using RNA-based microarray techniques in tissue samples from prostate cancer patients [10]. XMRV was detected predominantly in patients who are homozygous for the QQ allele at R462Q in the RNaseL gene, which results in a reduced RNaseL activity and therefore in a diminished IFN-based antiviral defense. Later studies showed XMRV to be an infectious virus for human prostate-derived cells and to be sensitive to RNaseL-mediated inhibition of replication by IFN-β [9]. The question of whether carcinogenic transformation renders the prostate epithelia cells susceptible to XMRV infection as a bystander virus or whether XMRV is itself a prostate cancer causing agent has not yet been addressed. It was very recently shown that XMRV could be detected in 22Rv1 prostate carcinoma cells originally derived from a primary prostatic carcinoma [17]. This observation further highlights the need to clarify the participation of XMRV in the etiology of human prostate carcinomas.

As known for many years in other cancers, e.g. HPV in cervical carcinoma or other cancers (reviewed by zur Hausen, 2009 [18]), infectious agents causing inflammatory (precancerous) lesions are suspected to be involved in the pathogenesis of prostate carcinoma [19,20]. An increased susceptibility of prostate epithelia cells to infection with RNA-viruses as a result of the impaired function of RNaseL, resulting in proliferative inflammatory atrophy (PIA), could be an intriguing scenario. These focal areas of epithelial atrophy are presumed to be precursors of prostatic intraepithelial neoplasia and prostate cancer [21]. A small number of other studies during the last ten years attempting to demonstrate a role for viral infections in the development of PCa have yielded rather inconclusive data [22-26].

If a real correlation between viral infection and prostate cancer exists, new therapeutic or even prophylactic treatments against the development of PCa could be developed by targeting, for example, viral antigens. In this respect, a recent observation that radiolabelled therapeutic monoclonal antibodies specific for HPV or HBV proteins can inhibit subcutaneous tumor development *in vivo *by cells expressing these antigens [27] is of particular interest.

In the present study, we report the testing of 589 DNA and RNA samples from sporadic prostate cancer patients for the RNaseL genotype and for XMRV sequences. Although 76 of our samples (12.9%) displayed the "susceptibility" QQ genotype, consistent with the frequency given in the literature, no XMRV-specific sequences were detected in either the RNA or the DNA from the prostate tumor samples. Given the ratio of approximately 40% positive cases harboring the QQ genotype in the study population of Urisman *et al*. [10], one would have expected at least 30 XMRV positive specimens amongst our 76 RNaseL QQ-allele samples.

At least two other studies have looked for XMRV at the nucleic acid level, albeit with a much smaller sample groups. Fischer and coworkers [11] studied material from 105 German patients with sporadic prostate cancer and found only one individual positive for XMRV by nested RT-PCR, but this individual did not display the QQ RNaseL genotype. Another study carried out in Ireland investigated 139 PCa patients. In 7 QQ patients and two heterozygous RQ samples, no XMRV sequences were detected [12].

It should be mentioned that this study cannot completely rule out the possibility of an infection with another gammaretrovirus in these patients. The design of our PCR approach was done in such a way that one primer pair (In-For/In-Rev) binds to conserved regions, allowing amplification of various MLV types including AKV MLV (J01998), MLV DG-75 (AF221065), MoMuLV (NC_001501), MTCR (NC_001702), MCF 1233 MLV (U13766), and Rauscher MuLV (NC_001819). In this PCR setup a specific signal was obtained with the mouse tail DNA as template, indicating that endogenous MLVs were detected. As additional controls we tested the cell lines 22Rv1 (XMRV positive [17]) and DU145 (XMRV negative [9]). As expected, 22Rv1 was found to be strongly positive for RNA transcripts and for provirus (with In-For/Deletion-Rev primers), while DU145 was negative in both PCR approaches (data not shown).

We also tested 146 sera samples for XMRV antibodies and found none of them to be positive in ELISA or Western blot analyses. The recombinant XMRV proteins that were used reacted positively with sera from immunized mice. As XMRV is closely related to other murine leukemia viruses and therefore immunogenic in mammalian hosts [28], an infection which allows the virus to spread to the stroma cells should induce a humoral immune response. The analysis of sera from prostate cancer patients for antibodies could therefore offer a rapid and valid screening method to investigate the involvement of a virus. Obviously the determination of sensitivity and specificity of these ELISA assays is to a certain degree limited, due to the lack of a human anti-XMRV positive control antibody. Nevertheless, the mouse sera were used to demonstrate the suitability of the recombinant antigens as ELISA antigens, even though the titration cannot be used to determine the amount of antibodies in the human sera samples. The failure to detect XMRV proviruses or transcripts in the 30 cases where DNA, RNA and sera samples were all available, is consistent with the negative ELISA results. It is theoretically possible that the tumor environment itself compromises the immune system and inhibits the antibody response to the tumor-associated viral antigens. This seems unlikely since animal studies have demonstrated that tumor diseases do not dramatically suppress systemic immunity [29]. There was a certain degree of background reactivity to the recombinant Gag proteins, as was also seen in an ELISA using a lysate of ultracentrifuge-concentrated virus as antigen (data not shown). Difficulties with background signals in testing human sera for reactivity to MLV-derived antigens are well known when using whole virion particles as antigen [30], but this also occurs to a lesser extent when using recombinant proteins [28]. In general, there was a higher background reactivity against Gag in our 146 PCa and healthy control sera tested; and one serum reacted strongly to the pr65 protein. Upon further testing in Western blot and immunfluorescence assay, this serum showed no specificity for XMRV. It might be possible that antibodies directed against the transmembrane protein p15E were missed due to our choice of the gp70 and the pr65 antigen as targets. In other human retrovirus infections, HIV and HTLV antibodies against this region are detectable. Therefore, it should also be mentioned that before the serological assays using XMRV proteins were established all serum samples were screened for cross-reactivity with recombinant gp70, p15E and p27 [31,32] of another gammaretrovirus, the porcine endogenous retrovirus (PERV). All sera were found to be negative for any of these targets despite the obvious sequence homology of XMRV and PERV in the ectodomain of p15E and certain conserved regions in gp70 and p27. Regarding this point, it is also of interest that Furuta *et al*. [33], recently reported the detection by Western blot of antibodies specific for the XMRV Gag protein in blood bank samples from prostate cancer patients and healthy donors, but no Env-specific antibodies.

## Conclusion

In summary, we demonstrate in a large cohort of more than 500 German prostate cancer patients with a median age of 63 years and various stages of disease no evidence for infection by the recently discovered gammaretrovirus XMRV. This result possibly suggests that the rather restricted geographic incidence of XMRV infections, and the epidemiology of XMRV in the United States should therefore be studied closely. In addition, the oncogenic potential of the virus should be thoroughly investigated to exclude (or confirm) this viral infection as a possible trigger for the development of prostate cancer.

## Abbreviations

XMRV: xenotropic murine leukemia virus-related virus; PCa: prostate cancer; MLV: murine leukemia virus; pr: precursorprotein; gp: glycoprotein; SNP: single nucleotide polymorphism; nt: nucleotide;

## Competing interests

The authors declare that they have no competing interests.

## Authors' contributions

OH carried out the molecular studies and drafted the manuscript. HK carried out the patient's sampling and preparation and drafted together with OH the manuscript. PB, LN and NaB carried out the generation and evaluation of antisera and corrected the manuscript. JD carried out the additional screening against related retroviruses. KM, RK and NB conceived of the study, and participated in its design and coordination. All authors read and approved the final manuscript.
